# Brain Circulation during Panic Attack: A Transcranial Doppler Study with Clomipramine Challenge

**DOI:** 10.1155/2014/296862

**Published:** 2014-03-16

**Authors:** Francesco Rotella, Marinella Marinoni, Francesca Lejeune, Fabiana Alari, Daniela Depinesi, Fiammetta Cosci, Carlo Faravelli

**Affiliations:** ^1^Psychiatry Unit, Careggi University Hospital, Largo Brambilla 3, 50100 Florence, Italy; ^2^Neurology Unit, Careggi University Hospital, Largo Brambilla 3, 50100 Florence, Italy; ^3^Section of Psychology and Psychiatry, Department of Health Sciences, University of Florence, Viale Pieraccini 6, 50139 Florence, Italy

## Abstract

*Introduction*. Cerebral blood flow has been well studied in patients with panic disorder, but only few studies analyzed the mechanisms underlying the onset of a panic attack. The aim of the present study was to monitor the cerebral hemodynamics modifications during a panic attack. *Materials and Methods*. 10 panic disorder patients with recent onset, fully drug naïve, were compared to 13 patients with panic disorder with a previous history of treatment and to 14 controls. A continuous bilateral monitoring of mean flow velocities in right and left middle cerebral arteries was performed by transcranial Doppler. Clomipramine was chosen as challenge. *Results*. Eight out of 10 patients drug naïve and 6 control subjects out of 13 had a full blown panic attack during the test, whereas none of the patients with a history of treatment panicked. The occurrence of a panic attack was accompanied by a rapid decrease of flow velocities in both right and left middle cerebral arteries. *Discussion*. The bilateral acute decrease of mean flow velocity during a panic attack suggests the vasoconstriction of the microcirculation of deep brain structures perfused by middle cerebral arteries and involved in the so-called “fear circuitry,” thus suggesting that cerebral homeostatic dysfunctions seem to have a key role in the onset of a panic attack.

## 1. Introduction

Cerebral blood flow (CBF) abnormalities have been reported in panic disorder (PD). These CBF modifications have been studied using different imaging techniques: positron emission tomography (PET) [1–3], single photon emission computed tomography (SPECT) [4, 5], and functional magnetic resonance imaging (fMRI) [6, 7], without a clear identification of the structures involved. In these studies the central hypothesis was that blood flow is coupled to brain activation/deactivation and, therefore, authors chose to use techniques with a greater spatial resolution in order to map these events in the brain at the expense of temporal resolution.

Few studies [8–11] could observe PD patients during a panic attack (PA) and, of these, only two [10, 11] managed to describe cerebral activity, using fMRI. Pfleiderer et al. [10] found a significant increase of activity in the right amygdala during the onset of a spontaneous PA, whereas Dresler and colleagues [11] observed that the neuronal dynamics of the structures involved in the fear network (i.e., amygdala, insula, and prefrontal cortex) mirrored the description of the attack made by a patient that experienced a full blown, spontaneous PA while performing fMRI.

The study of cerebral hemodynamics during the onset of a PA would be crucial as a certain evidence of different mechanisms underlying PAs is emerging. However, studying cerebral hemodynamics during a PA is difficult because of three factors: (a) it is difficult to pinpoint an occurring PA; (b) the techniques commonly used to assess brain circulation are not sensitive to the real time variations; (c) the challenges commonly used to elicit a PA influence the brain circulation.

In order to observe unexpected PAs in a clinical setting and to study their pathophysiology, several panicogenic agents able to provoke PAs in a controlled laboratory setting have been proposed and widely used: sodium lactate [12–14], carbon dioxide at different concentrations [15–21], cholecystokinin [22–27], and serotoninergic agents [28–32]. All these challenges, however, produce intrinsic effects on cerebral circulation and are therefore inappropriate to study CBF modifications during a PA.

The idea to use a serotonin reuptake inhibitor (SRI) as a challenge was generated by the clinical observation of the so-called biphasic effect, with initial exacerbation of anxiety, which most patients experience during the first days of treatment with a SRI [33–37]. Two SRI are available for intravenous administration in Europe, namely, citalopram and clomipramine (CMI). To our knowledge, only CMI has already been used as a challenge. The challenge with CMI induces neuroendocrine modifications, does not increase noradrenergic activity, and is sensitive to the effect of serotonin receptors antagonist [38, 39]. CMI, on the other hand, is the agent with less interference with CBF, compared to the above-mentioned panicogenic agents [40].

As already stated, PET and SPECT have a low sensibility in detecting rapid CBF modifications. fMRI, although very sensitive for cortical areas, is less accurate in detecting deep brain structures modifications and is expensive and generally less acceptable to phobic patients.

The transcranial Doppler (TCD) ultrasound technique is not invasive and allows, with a pulsed emission of low-frequency ultrasounds, the measurement of mean flow velocity (MFV) in the main intracranic arteries [41]. Although not very sensitive in focusing on specific areas, TCD is particularly suited to assess rapid flow velocity variations in real time, in response to challenges. Furthermore TCD is inexpensive, easy to perform, and totally safe and allows a continuous and bilateral monitoring of arteries.

CBF abnormalities have been reported using TCD in PD patients. To our knowledge, however, two are the studies that performed TCD during a PA [8, 9]. Fontaine et al. [9] found a bilateral rapid increase of blood flow velocity in the MCAs, after sodium lactate infusion, greater in patients with PD compared with healthy controls, whereas Alkin, using the 35% carbon dioxide challenge, observed an increase in the basilar artery blood flow velocity [8]. In these studies, however, continuous TCD monitoring was not performed.

While studying PD patients outside the PA, Owega et al. [42] found the same variations described above, associated with significant flow acceleration in the middle and anterior cerebral arteries and in the left posterior cerebral artery. In a different study, PD patients showed asymmetric variations with a higher mean blood flow velocity in the right MCA [43]. A reduction of right MCA mean flow velocity in patients with acute and remitted PD was also reported following tilting to the upright position [44].

In this framework, our hypothesis was that the onset of a PA is accompanied by rapid blood flow velocities modifications in the MCAs, as these arteries perfuse the deep brain structures involved in the fear network. Thus, the aim of the present study was to monitor the cerebral hemodynamic of MCAs during the onset of a full blown PA, using TCD. CMI was chosen as challenge to provoke PAs in a clinical setting for its slight interference with CBF and because it is safe and ethically acceptable, being a drug used in the treatment for PD.

## 2. Materials and Methodology

Eleven patients meeting the diagnosis of PD according to Diagnostic and Statistical Manual of Mental Disorders IV Edition (DSM IV) [45] with recent onset (i.e., within 3 months of the first PA), fully drug naïve, were compared with 13 patients suffering from PD for longer time, all with a history of previous treatment, but drug-free for at least two weeks, and with 14 healthy volunteers.

Exclusion criteria were pregnancy and nursing, menopause, neurological diseases, mental retardation, epilepsy, migraine, hypertension, substance abuse, and use of psychotropic drugs during the two weeks before the test.

All subjects were asked to refrain from smoking and drinking coffee during the three hours before the study and to follow a low monoamines diet in the preceding three days. All the tests were performed in the morning to standardize the environmental conditions.

Each participant was given detailed information on the examination and gave their written consent and the protocol was approved by the local ethic committee.

All the subjects received a careful medical examination and were interviewed by the Structured Clinical Interview for DSM (SCID) [46].

The test, which consisted of four different phases, was performed as a single-blind study. Each subject was informed that he would have been injected, in random sequence, placebo, and CMI.

A commercially available 2 MHz pulsed-wave TCD unit (MultiDop X4, DWL Compumedics) continuously and simultaneously monitored mean CBF velocity on MCA bilaterally by using the Aaslid technique [41]. Two TCD probes were fixed over left and right temporal windows by Spencer helmet.

Starting from an insonation depth of 50 mm, depths and angles of insonation were adjusted to get the best Doppler signal of the M1 segment for left and right MCAs.

All the participants were informed of the possibility that they could experience panic symptoms during the test and were encouraged to continue the test whenever possible. They were asked not to speak during the monitoring and to signal the onset of panic/discomfort symptoms just moving the right hand.

The first phase (baseline) consisted in Doppler monitoring (10 minutes), at rest, until steady MCAs velocities were attained. During the second phase (placebo) a placebo infusion of 100 mL of saline solution i.v. was administered (14 minutes). The third phase (CMI) consisted in the infusion of 12.5 mg of CMI, administered i.v. in 100 mL of saline solution (15 minutes). Finally, during the fourth phase (10 minutes) (wash), CMI infusion was stopped, continuing Doppler monitoring until reestablishment of baseline conditions. The TCD monitoring was conducted during each phase of the test.

Before wearing the helmet for TCD monitoring, each subject completed the following instruments: panic symptom list (PLS), visual scale of anxiety (VAAS) [47], and state trait anxiety inventory (STAI) [48]. These scales were also administered before the commencement of the procedure and at the end of each phase of the test to evaluate anxiety and panic symptoms.

Heart and breathing rates were continuously monitored using a Fukuda Denshi Dynascope device during the entire examination. The device did not allow data recording. Blood pressure was measured at the beginning and the end of each phase of the test.

The test has always been performed by two investigators in order to continuously control modifications of Doppler signal and respiratory and heart frequencies.

### 2.1. Offline Analyses

At the end of the test several offline analyses were performed. For each subject average values of MFV, expressed by cm/sec, were calculated for each phase of the test. The third phase was then theoretically split into two subphases (CMI_1_ and CMI_2_) of 7.5 minutes each, taking into account the latency of the drug effect.

The highest MFV variation during the whole period of the test was then measured for each participant.

### 2.2. Statistics

*t*-test for independent samples, chi square test, and one-way ANOVA were performed when appropriate. The criteria for PA were occurrence of at least 4 symptoms described in the PSL and VAAS increase of at least 25 mm.

The Statistical Package for the Social Sciences for Windows SPSS (IBM, 2011) version 20.0 was used for data analysis and results were considered significant when *P* values were ≤0.05.

## 3. Results and Discussion

### 3.1. Results

The group of patients with PD with recent onset was composed of 5 males and 6 females, with a mean age of 26.1 ± 8.2 years; the group of patients suffering from PD for longer time was composed of 6 males and 7 females, with a mean age of 32.6 ± 9.0 years; the control subjects were 5 males and 8 females, with a mean age of 28.1 ± 3.4 years. No statistically significant difference was observed among the three groups for gender and age.

One PD patient with recent onset and drug naïve reported great discomfort four minutes after wearing the Spencer helmet, became restless, and asked to stop the Doppler monitoring. All the other subjects managed to complete the four phases of the test. The final sample eligible for statistical analysis of cerebral hemodinamic was therefore composed of 10 PD patients with recent onset, 13 PD patients with a previous history of treatment, and 14 controls.

Eight out of 10 PD patients who were drug naïve and 6 out of 13 controls had a PA during the test, whereas none of the patients with PD with a previous history of treatment panicked. The comparisons were statistically significant for drug-naïve PD versus PD with previous treatment (chi square = 15.9, df = 1; *P* = 0.0001) and for healthy controls versus PD with previous treatment (chi square = 7.2, df = 1; *P* = 0.0074), while the number of panickers failed to distinguish significantly drug-naïve PD from controls (chi square = 2.71; *P* = 0.09).

Of the 8 patients with PD that had a PA, 1 had it during the first phase of the test, 2 during the placebo infusion, and 5 during the CMI challenge. All the 6 controls had the onset of the PA during the CMI infusion.

The mean MFVs for each group were compared for each phase of the test and no statistically significant difference was found (data not shown).

As there was no specific response to CMI for diagnosis, we split the sample into two groups: those who had panic during the test (panickers) and those who had not (nonpanickers).

**Figure 1:**
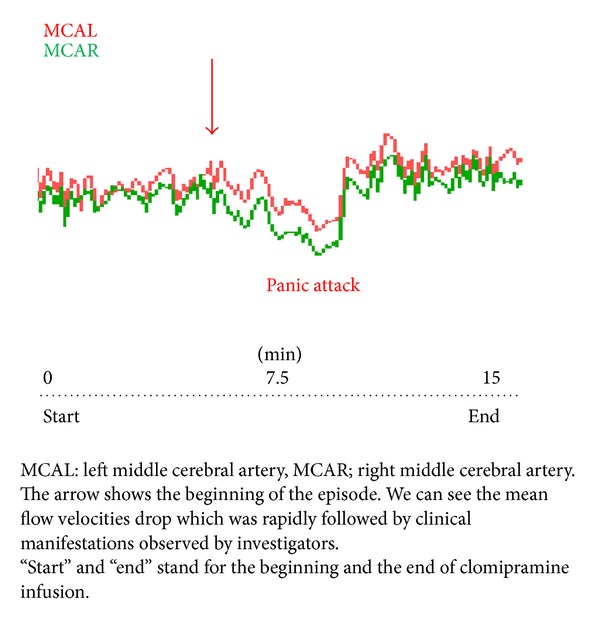
Doppler signal during clomipramine infusion: typical mean flow velocities drop during the onset of a panic attack.

At the first direct visual observation of the blood velocity graphics, the sudden drop of MFV at both the right and left MCAs was clearly visible during a PA (Figure 1).

The average MFVs during each different phase of the test were compared between panickers and nonpanickers and no statistically significant difference was found during any phase of the test, for both right and left MCAs (Table 1).

The highest MFV variations clearly distinguished panickers from nonpanickers, in the right as well as in the left MCA, both using absolute and relative (%MFV variations from baseline) values (Table 2).

Comparing average MFV values during the CMI infusion period to those obtained during baseline conditions, statistically significant differences were found when comparing panickers to nonpanickers when panickers were compared to nonpanickers. For right MCA, we found an average MFV difference of −1.49 ± 3.44 cm/sec in panickers and of 0.90 ± 3.02 cm/sec for nonpanickers (*P* < 0.05). For left MCA we found a mean MFV difference of −2.25 ± 5.17 cm/sec in panickers and of 0.57 ± 2.58 cm/sec in nonpanickers (*P* < 0.05).

### 3.2. Discussion

The basic finding of this research is a significant drop of MFV in both right and left MCAs during the onset of a PA performing a provocative challenge with CMI. CMI challenge has not been frequently used in literature as a provocative test to induce PAs in a clinical setting [38]. In our study, CMI did not seem to be a challenge specific to PD, as it induced PAs in both PD patients and control subjects.

In contrast with other results [42] that reported increased bilateral middle and anterior cerebral artery and left posterior cerebral artery velocity at rest, our findings showed no difference in MCAs' MFV, between the three groups at baseline conditions.

It is notable that none of the subjects with a previous lifetime treatment with antidepressants, even if drug-free when performing the test, reported a PA during the challenge. Whether this is due to a sort of in vivo exposure to antidepressant side effect, cognitively mediated, or to a long lasting decreased sensitivity to the anxiogenic effect of acute administration of serotoninergic agents cannot be ascertained.

Although TCD actually measures the blood flow velocity in the large arteries, it is commonly accepted that a reduction of flow velocity is associated with the vasoconstriction of the microcirculation supplied by that artery. The bilateral acute decrease of MFV during a PA found in this study suggests the vasoconstriction of the microcirculation of deep brain structures perfused by MCAs and involved in the neuroanatomy of fear [49, 50].

This finding should not be secondary to the effects of CMI challenge. This statement is confirmed by two observations. We had the opportunity to study MFV pattern of three patients who experienced a PA outside the period of CMI infusion (one during baseline conditions and two during placebo infusion) and these subjects showed the same pattern of response of those who panicked during CMI infusion. On the other hand, none of the subjects who did not have a PA showed any MFV modification while performing the challenge. Some studies reported that severe anxiety decreases CBF [51, 52]. The observation that this kind of response is specific for acute anxiety and not for PD is confirmed by the fact that in our study no difference in MFV modifications was found when comparing PD subjects with controls.

Our data seem to be consistent with the hypothesis of an autonomic nervous system dysregulation involved in the pathogenesis of PD. Several lines of evidence suggest this relationship. First of all, most of the somatic symptoms of panic attacks are mediated by the autonomic nervous system.

Many studies have reported a variety of autonomic dysfunctions in patients with PD (e.g., [53, 54]); these include excessive autonomic nervous system activation and reactivity, functional modifications of the parasympathetic and sympathetic systems, and changes in *α* and *β* adrenoreceptor function. However, other studies did not confirm the role of the autonomic nervous system in PD [55, 56].

It could be hypothesized that the MCV modifications observed in the present study could be due to hyperventilation-induced hypocapnia. In fact, the relationship between the decrease in CBF velocities and panic-induced hyperventilation/hypocapnia is well established (for a review see [57]). In our study, PaCO_2 _was not measured and the device used to continuously monitor respiratory rates did not allow data recording. This represents the main limitation of the work, as it was not possible to perform multivariate statistical analyses adjusted for these variables. However, although factors such as the respiratory volume or the presence of “shallow breathing” may influence the PaCO_2_, thus making the respiratory rate a not perfect correlate of PaCO_2_, the continuous qualitative observation of heart and respiratory rates during the test suggested for us that MFV drop occurs immediately before the onset of tachycardia and hyperventilation in every subject that panicked during the test. This suggests a strong association between microcirculation vasoconstriction and the onset of a PA, regardless of the influence of other possible confounding factors.

## 4. Conclusion

Despite the above limitations, the present study suggests that cerebral homeostatic dysfunctions may have a key role in the onset of a PA. These data support the hypothesis that an autonomic dysregulation can be the trigger of panic symptoms occurrence [44, 53, 54]. However, replication studies examining the PaCO_2_, or at least the respiratory rate, are warranted in order to better understand the possible etiopathogenetic mechanisms underlying panic attacks.

## Figures and Tables

**Table 1:** Comparison of average MFV values in the four phases of the test (CMI values split also into CMI_1_ and CMI_2_) for both left and right middle cerebral arteries, between panickers and nonpanickers.

	Mean MFV (cm/sec)		*P*
	Panickers (*n* = 14)	Nonpanickers (*n* = 23)	
Baseline			
RMCA	67.2	67.2	0.99
LMCA	70.5	67.5	0.53
Placebo			
RMCA	67.1	67.1	0.99
LMCA	71.5	65.4	0.21
CMI_1_			
RMCA	67.2	67.3	0.97
LMCA	70.3	65.3	0.33
CMI_2_			
RMCA	63.6	68.9	0.30
LMCA	67.9	66.8	0.84
CMI			
RMCA	65.6	68.0	0.64
LMCA	69.3	65.9	0.52
Wash			
RMCA	68.3	68.2	0.98
LMCA	70.4	65.6	0.48

MFV: mean flow velocity; RMCA: right middle cerebral artery; LMCA: left middle cerebral artery; CMI: clomipramine.

**Table 2:** Comparison between panickers and nonpanickers for highest MFV variations in both left and right middle cerebral arteries.

	Panickers (*n* = 14)	Nonpanickers (*n* = 23)	*P*
Highest MFV variation (cm/sec)			
RMCA	−16.92 ± 9.96	−6.45 ± 5.40	<0.001
LMCA	−15.41 ± 8.64	−6.14 ± 4.95	<0.001
Highest % MFV variation			
RMCA	−24.31 ± 14.12	−10.31 ± 7.87	0.001
LMCA	−20.56 ± 11.44	−9.61 ± 6.91	0.001

Data expressed as mean ± standard deviation.

MFV: mean flow velocity; RMCA: right middle cerebral artery; LMCA: left middle cerebral artery.

## References

[1] Nordahl TE, Semple WE, Gross M (1990). Cerebral glucose metabolic differences in patients with panic disorder. *Neuropsychopharmacology*.

[2] Reiman EM, Raichle ME, Robins E (1986). The application of positron emission tomography to the study of panic disorder. *American Journal of Psychiatry*.

[3] Sakai Y, Kumano H, Nishikawa M (2005). Cerebral glucose metabolism associated with a fear network in panic disorder. *NeuroReport*.

[4] de Cristofaro MTR, Sessarego A, Pupi A, Biondi F, Faravelli C (1993). Brain perfusion abnormalities in drug-naive, lactate-sensitive panic patients: a SPECT study. *Biological Psychiatry*.

[5] Eren I, Tükel R, Polat A, Karaman R, Ünal S (2003). Evaluation of regional cerebral blood flow changes in panic disorder with Tc99m-HMPAO SPECT. *Psychiatry Research: Neuroimaging*.

[6] Bystritsky A, Pontillo D, Powers M, Sabb FW, Craske MG, Bookheimer SY (2001). Functional MRI changes during panic anticipation and imagery exposure. *NeuroReport*.

[7] Maddock RJ, Buonocore MH, Kile SJ, Garrett AS (2003). Brain regions showing increased activation by threat-related words in panic disorder. *NeuroReport*.

[8] Alkin T, Tural Ü, Onur E, Öztürk V, Monkul ES, Kutluk K (2007). Basilar artery blood flow velocity changes in patients with panic disorder following 35% carbon dioxide challenge. *Progress in Neuro-Psychopharmacology and Biological Psychiatry*.

[9] Fontaine S, Ontiveros A, Fontaine R, Elie R (1991). Panic disorder: vascular evaluation with transcranial Doppler ultrasonography. *Canadian Association of Radiologists Journal*.

[10] Pfleiderer B, Zinkirciran S, Arolt V, Heindel W, Deckert J, Domschke K (2007). fMRI amygdala activation during a spontaneous panic attack in a patient with panic disorder. *World Journal of Biological Psychiatry*.

[11] Dresler T, Hahn T, Plichta MM (2011). Neural correlates of spontaneous panic attacks. *Journal of Neural Transmission*.

[12] Stewart RS, Devous MD, Rush AJ, Lane L, Bonte FJ (1988). Cerebral blood flow changes during sodium-lactate-induced panic attacks. *American Journal of Psychiatry*.

[13] Kelly D, Mitchell-Heggs N, Sherman D (1971). Anxiety and the effects of sodium lactate assessed clinically and physiologically. *British Journal of Psychiatry*.

[14] Liebowitz MR, Fyer AJ, Gorman JM (1984). Lactate provocation of panic attacks. I. Clinical and behavioral findings. *Archives of General Psychiatry*.

[15] Fyer MR, Uy J, Martinez J (1987). CO_2_ challenge of patients with panic disorder. *American Journal of Psychiatry*.

[16] Gorman JM, Askanazi J, Liebowitz MR (1984). Response to hyperventilation in a group of patients with panic disorder. *American Journal of Psychiatry*.

[17] Gorman JM, Papp LA, Martinez J (1990). High-dose carbon dioxide challenge test in anxiety disorder patients. *Biological Psychiatry*.

[18] Griez EJL, Lousberg H, van den Hout MA, van der Molen GM (1987). CO_2_ vulnerability in panic disorder. *Psychiatry Research*.

[19] Griez E, de Loof C, Pols H, Zandbergen J, Lousberg H (1990). Specific sensitivity of patients with panic attacks to carbon dioxide inhalation. *Psychiatry Research*.

[20] Griez E, Zandbergen J, Pols H, de Loof C (1990). Response to 35% CO_2_ as a marker of panic in severe anxiety. *American Journal of Psychiatry*.

[21] Freire RC, Lopes FL, Valença AM (2008). Panic disorder respiratory subtype: a comparison between responses to hyperventilation and CO_2_ challenge tests. *Psychiatry Research*.

[22] Bourin M, Malinge M, Guitton B (1995). Provocative agents in panic disorder. *Therapie*.

[23] Bradwejn J, Koszycki D, Payeur R, Bourin M, Borthwick H (1992). Replication of action of cholecystokinin tetrapeptide in panic disorder: clinical and behavioral findings. *American Journal of Psychiatry*.

[24] de Montigny C (1989). Cholecystokinin tetrapeptide induces panic-like attacks in healthy volunteers. Preliminary findings. *Archives of General Psychiatry*.

[25] Eser D, Schüle C, Baghai T (2007). Evaluation of the CCK-4 model as a challenge paradigm in a population of healthy volunteers within a proof-of-concept study. *Psychopharmacology*.

[26] Schunck T, Erb G, Mathis A (2006). Functional magnetic resonance imaging characterization of CCK-4-induced panic attack and subsequent anticipatory anxiety. *NeuroImage*.

[27] Javanmard M, Shlik J, Kennedy SH, Vaccarino FJ, Houle S, Bradwejn J (1999). Neuroanatomic correlates of CCK-4-induced panic attacks in healthy humans: a comparison of two time points. *Biological Psychiatry*.

[28] Apostolopoulos M, Judd FK, Burrows GD, Norman TR (1993). Prolactin response to dl-fenfluramine in panic disorder. *Psychoneuroendocrinology*.

[29] Kahn RS, Westenberg HG, Verhoeven WM, Gispen-de Wied CC, Kamerbeek WD (1987). Effect of a serotonin precursor and uptake inhibitor in anxiety disorders; a double-blind comparison of 5-hydroxytryptophan, clomipramine and placebo. *International Clinical Psychopharmacology*.

[30] Kahn RS, Asnis GM, Wetzler S, van Praag HM (1988). Neuroendocrine evidence for serotonin receptor hypersenstivity in panic disorder. *Psychopharmacology*.

[31] Targum SD (1990). Differential responses to anxiogenic challenge studies in patients with major depressive disorder and panic disorder. *Biological Psychiatry*.

[32] van der Wee NJA, Fiselier J, van Megen HJGM, Westenberg HGM (2004). Behavioural effects of rapid intravenous administration of meta-chlorophenylpiperazine in patients with panic disorder and controls. *European Neuropsychopharmacology*.

[33] Catalano G, Hakala SM, Catalano MC (2000). Sertraline-induced panic attacks. *Clinical Neuropharmacology*.

[34] Spigset O (1999). Adverse reactions of selective serotonin reuptake inhibitors: reports from a spontaneous reporting system. *Drug Safety*.

[35] Altshuler LL (1994). Fluoxetine-associated panic attacks. *Journal of Clinical Psychopharmacology*.

[36] Zinner SH (1994). Panic attacks precipitated by sertraline. *American Journal of Psychiatry*.

[37] Saran A, Halaris A (1989). Panic attack precipitated by fluoxetine. *The Journal of Neuropsychiatry and Clinical Neurosciences*.

[38] George DT, Nutt DJ, Rawlings RR (1995). Behavioral and endocrine responses to clomipramine in panic disorder patients with or without alcoholism. *Biological Psychiatry*.

[39] Golden RN, Hsiao J, Lane E, Hicks R, Rogers S, Potter WZ (1989). The effects of intravenous clomipramine on neurohormones in normal subjects. *Journal of Clinical Endocrinology and Metabolism*.

[40] Buchweitz E, Roffman M, Weiss HR (1984). Immediate vs. long-term desmethylimipramine or chlorimipramine: effects on regional cerebral blood flow. *European Journal of Pharmacology*.

[41] Aaslid R, Markwalder TM, Nornes H (1982). Noninvasive transcranial Doppler ultrasound recording of flow velocity in basal cerebral arteries. *Journal of Neurosurgery*.

[42] Owega A, Sabri O, Klingelhöfer J, Albers M (2001). Cerebral blood flow velocity in untreated panic disorder patients: a transcranial Doppler ultrasonography study. *Biological Psychiatry*.

[43] Cerisoli M, Amorf M, Campanile S, Scardovi F, Borromei A, Alvisi C (1996). Evaluating CBF velocity changes with transcranial Doppler ultrasound. *American Journal of Psychiatry*.

[44] Faravelli C, Marinoni M, Spiti R (1997). Abnormal brain hemodynamic responses during passive orthostatic challenge in panic disorder. *American Journal of Psychiatry*.

[45] American Psychiatric Association (1994). *Diagnostic and Statistical Manual of Mental Disorders*.

[46] First MB, Spitzer RL, Gibbon M, Williams JBW (1997). *SCID-I: Structured Clinical Interview for DSM-IV Axis I Disorders*.

[47] Hornblow AR, Kidson MA (1976). The visual analogue scale for anxiety: a validation study. *Australian and New Zealand Journal of Psychiatry*.

[48] Spielberger CD, Gorsuch RL, Lusene RE (1970). *STAI Manual for the State-Trait Anxiety Inventory*.

[49] Goddard AW, Charney DS (1997). Toward an integrated neurobiology of panic disorder. *Journal of Clinical Psychiatry*.

[50] Charney DS (2003). Neuroanatomical circuits modulating fear and anxiety behaviors. *Acta Psychiatrica Scandinavica, Supplement*.

[51] Mathew RJ, Wilson WH, Humphreys D, Lowe JV, Wiethe KE (1997). Cerebral vasodilation and vasoconstriction associated with acute anxiety. *Biological Psychiatry*.

[52] Zohar J, Insel TR, Berman KF, Foa EB, Hill JL, Weinberger DR (1989). Anxiety and cerebral blood flow during behavioral challenge. Dissociation of central from peripheral and subjective measures. *Archives of General Psychiatry*.

[53] Stein MB, Tancer ME, Uhde TW (1992). Heart rate and plasma norepinephrine responsivity to orthostatic challenge in anxiety disorders: comparison of patients with panic disorder and social phobia and normal control subjects. *Archives of General Psychiatry*.

[54] Hoehn-Saric R, McLeod DR (1988). The peripheral sympathetic nervous system. Its role in normal and pathologic anxiety. *Psychiatric Clinics of North America*.

[55] Stein MB, Asmundson GJG (1994). Autonomic function in panic disorder: cardiorespiratory and plasma catecholamine responsivity to multiple challenges of the autonomic nervous system. *Biological Psychiatry*.

[56] Asmundson GJG, Stein MB (1994). Vagal attenuation in panic disorder: an assessment of parasympathetic nervous system function and subjective reactivity to respiratory manipulations. *Psychosomatic Medicine*.

[57] Dratcu L (2000). Panic, hyperventilation and perpetuation of anxiety. *Progress in Neuro-Psychopharmacology and Biological Psychiatry*.

